# The Effect of High-Frequency Electrical Stimulation of Bilateral Nucleus Accumbens on the Behavior of Morphine-Induced Conditioned Place Preference Rats at Extinction and Reinstatement Phases

**DOI:** 10.1155/2020/8232809

**Published:** 2020-10-12

**Authors:** Chunhui Yang, Yiqing Qiu, Xiaowu Hu, Jianchun Chen, Yina Wu, Xi Wu

**Affiliations:** Department of Neurosurgery, Changhai Hospital, Shanghai 200433, China

## Abstract

**Objective:**

To explore the optimal time points for deep brain stimulation (DBS) on the treatment of morphine addiction and its possible mechanisms by investigating how high-frequency stimulation (HFS) in bilateral nucleus accumbens (NAc) at different time points influences the addictive behaviors of rats with drug addiction.

**Methods:**

The rats were randomly divided into extinction stimulation group (*n* = 20) and postextinction stimulation group (*n* = 20). Ten rats in the extinction stimulation group were treated using 120 Hz HFS during extinction stage while another 10 rats with pseudostimulation were served as control group. The CPP scores were evaluated at the second day after intervention, with total 9 sections accomplished. The CPP scores were evaluated at the second day of the intervention. In the postextinction stimulation group, 120 Hz HFS was intervened during the postextinction stage in 10 experimental rats and pseudostimulation was performed in 10 control rats. Stimulation was performed for 7 days continuously, and a small dose of morphine was administrated to induce relapse after the postextinction period.

**Results:**

During the extinction phase, CPP scores after HFS were significantly higher. During the postextinction phase, relapse CPP scores after HFS were dramatically lower.

**Conclusion:**

HFS of bilateral NAc inhibits the extinction of addictive behavior during the extinction phase, and HFS during the postextinction period suppresses relapse of drug seeking behavior.

## 1. Background

Drug addiction is considered as an encephalopathy with recurrent attacks manifested with obsessive drug self-intake behaviors, which is characterized by nonmedical, long-term, and repeated increase of drug dosage [1]. It can be divided into four stages including addiction, extinction, postextinction, and relapse phases. Drug addiction is composed of physiological dependence in the extinction period and psychological dependence with strong obsession in the postextinction phase. Currently, physiological dependence can be managed by multiple approaches, while psychological dependence was difficult to be eliminated. The stressful events, drug-related cues, or drugs themselves can evoke the recall of previous addictive memory, leading to the repeated relapse and subsequent withdrawal failure, and thus developing effective therapies for drug addiction is urgently required.

Deep brain stimulation (DBS) is an approach to produce therapeutic efficacy through the modulation of neural circuits by implanting the electrode into the specific neural nucleus [2]. At present, DBS has been applied for the clinical treatment of multiple neurological diseases, including depression, obsession [3], Parkinson's disease (PD) [4, 5], and so on [6]. In PD patients, STN-DBS can attenuate the disease symptoms and the desire to take the dopaminergic drugs [7]. Recently, clinical trials have reported that electrical stimulation of nucleus accumbens (NAc) improves withdrawal of alcohol and smoking addictions in the patients [8].

The efficacy of DBS for drug addiction may be associated with the modulation of neural circuits of reward. The midbrain dopaminergic neurons were the key components in reward neural circuits [9]. The ventral tegmental area (VTA) and NAc were final common targets of various reward neural circuits in the brain. The general understanding is that the reward circuitry underlying addiction begins with the VTA and NAc is a major reward-related output of VTA circuitry. Thus, current evidence indicates that NAc may be a potential therapeutic target for addiction. The NAc is a part of ventral striatum, and thus it participates in the limbic information processing and is related to the enhancement of drug addiction [10, 11]. Therefore, NAc plays a key role in the pathogenesis of addiction [12, 13]. Recent studies have shown that electrical stimulation of bilateral NAc can effectively reduce the enhancement of drug addiction and the behavior and high-frequency stimulation can exert better therapeutic effects [14]. Thus, DBS is a novel treatment of drug addiction and NAc is an ideal target for drug addiction [12].

The effects of DBS on the various stages of drug addiction have been investigated and reported, while the influence of high-frequency stimulation (HFS) on the behavior of same drug at different stages has not been reported. The investigation of DBS for the intervention of drug seeking behavior at different stages is not only necessary to decipher the mechanisms underlying the effects of DBS on drug seeking behavior but also an objective requirement to correctly use DBS for the treatment of drug addiction in clinical practice. This study initially established the model of conditioned place preference (CPP) induced by morphine in rats, and then the HFS was delivered to bilateral NAc at the extinction and postextinction stages, respectively, to observe its effects on the addictive behavior of rats and to explore the mechanisms and optimal time points for the treatment of drug addiction using DBS.

## 2. Methods and Materials

### 2.1. Animals and Cannula Implantation

Forty Sprague-Dawley male rats with weight of 280–300 g were freely fed and selected for the experiments. The rats were provided by Shanghai Slack Experimental Animal Co. Ltd. (production license number: SCXK (Su) 2011-0003). All animal use protocols were reviewed and approved by the Institutional Animal Care and Use Committee (IACUC) of Shanghai Institute of Materia Medica (SIMM), which received the full accreditation of AAALAC in May 2011. All procedures were executed in accordance with the IACUC guidelines and polices. Before the behavior training, rats were anesthetized with intraperitoneal injection of 4% pentobarbital sodium (5 ml/kg). The implantation of cannula was performed using a stereotactic device during the operation. NAc core was located with reference to the stereotaxic atlas of rat brain according to the 5th edition rat brain atlas (the rat brain in stereotaxic coordinates 2004) (AP: 1.5 mm, ML: 2 mm, and DV: 6.4 mm). Dental cement was used for the cannula fixation and incision closure. After operation, rats were allowed to rehabilitate for 5 days. To prevent incision infection, they were subcutaneously injected with penicillin (400 kU/kg) for 3 days. After the end of the experiment, the rats were decapitated and their brains were quickly removed and frozen. Brain slices containing NAc were stained using H&E staining.

### 2.2. DBS Circuit Design

In the experiment, the bipolar stainless steel electrode was implanted and fixed. One end of the electrode line was connected to the connection port on the head of the rat. The other end was connected with an external pulse generator (Suzhou Jing Yu Medical Instrument Co., Ltd.) to form a complete stimulus circuit.

### 2.3. Conditioned Place Preference

The CPP video analysis system was acquired from Shanghai Jilin Software Technology Co., Ltd. It is made up of two video surveillance boxes. There are some channels in the middle for free shuttle of rats (the channel gate can be switched according to the requirement of the experiment). The surrounding walls of the left box are black and decorated with white cross streaks, and the bottom is a plate with cross iron streaks. The four walls of the right box are black and decorated with white longitudinal streaks, and the bottom grid iron plate can stimulate the rats with visual and touch stimulus. Both boxes were set up with an infrared video surveillance system. The rats were monitored to obtain the respective time when the rats stayed in the two boxes during test, and the time when the rats stayed in drug box was the CPP score.

The morphine-induced conditioned place preference model can be divided into four stages [15–18].

#### 2.3.1. Preconditioning Stage

Initially, the rats were allowed to rehabilitate for 5 days after the surgery and then were put in the CPP video box to adaptive training. During the adaption period, the gate of the channels was opened and rats can shuttle freely between the left and right boxes. The adaptive training was performed for 45 min per day for 3 days. On the fourth day, the stay time (preconditioning test) in both boxes within 900 s was recorded by the CPP video system. The nonpreferred box was selected as the morphine-treated box, and the stay time (s) for the nonpreferred box was preconditioning CPP score (if the rats stay in a single box for over 720 s, they will be rejected due to the existence of congenital positional preference, which will affect the experimental results).

#### 2.3.2. Conditioning Stage

The gates of the left and right box channels were closed. At 8:00–12:00 every day, the rats were subcutaneously injected with morphine hydrochloride [16, 19] (10 mg/kg, Shenyang First Pharmaceutical, batch number: 140505-1). After the injection, the rats were placed in the drug box for 45 min. At 14:00–18:00 of the same day, the rats were administrated with the same dose of saline and then were immediately placed in the nondrug box for 45 min. The training interval should be more than 6 hours, and the training was performed for 5 days continuously. On the sixth day, the postconditioning test was performed, and the channel gate was opened to test the stay time of rats in the drug box within 900 s. A paired *t*-test was used to compare the difference of preconditioning CPP score of the same rats. The results of a statistical significance (*P* < 0.05) was considered to successfully establish the morphine-induced CPP model.

#### 2.3.3. Extinction Stage

After the CPP model was successfully established, morphine was substituted with the same amount of saline to induce extinction with the same protocol. The next day, the extinction test was performed to obtain the stay time of the rats in the two boxes within 900 s, in which the time in the drug box was defined as extinction CPP score. The results were compared with the CPP scores of first and second phases using the paired *t*-test. When the extinction CPP score showed no difference to preconditioning CPP score (*P* > 0.05) and was statistically different from the postconditioning CPP score (*P* < 0.05), the morphine-induced CPP extinction was considered to be successfully established.

#### 2.3.4. Reinstatement Test

After the extinction, a priming dose of morphine (3 mg/kg) was administrated subcutaneously on the neck back of the rats, and the rats were placed in the boxes with free shuttle between the boxes. The stay time of the drug box (s) was defined as relapse CPP score and was compared with the preconditioning CPP score using the paired *t*-test. The comparison of the relapse scores between the two groups was performed by the independent two-sample *t*-test.

### 2.4. DBS Parameter Setting

**Figure 1:**
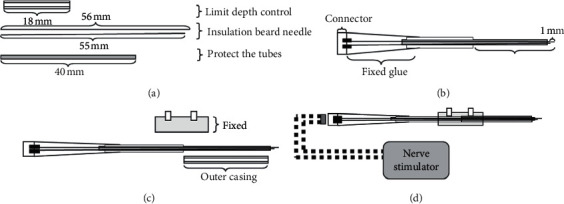
The diagram of implanted cannula and bipolar stainless steel electrode.

Based on previous stimulation studies [19–21], the current strength was set ranging from 0.2 mA to 60 mA [22–24]. The pulse width of 60 ms has been commonly used in HF-DBS in rats [20, 25–27]. A stimulation with frequency over 100 Hz belongs to high frequency range. In previous literature, a HFS was set ranging from 100 Hz to 160 Hz, so we set stimulation parameters as follows: stimulation frequency—120 Hz, wave width—60 ms, and stimulation intensity—0.3 mA. When stimulated, free-moving rats were put into the organic glass box (width: 30 cm, length: 50 cm, and height: 30 cm) and were stimulated for 30 min per day. In the experiment, no abnormal behaviors were observed in the rats, such as freezing, vocalizing, or jumping, which would be consistent with fear, pain, or discomfort (Figure 1).

### 2.5. Experimental Groups

**Figure 2:**
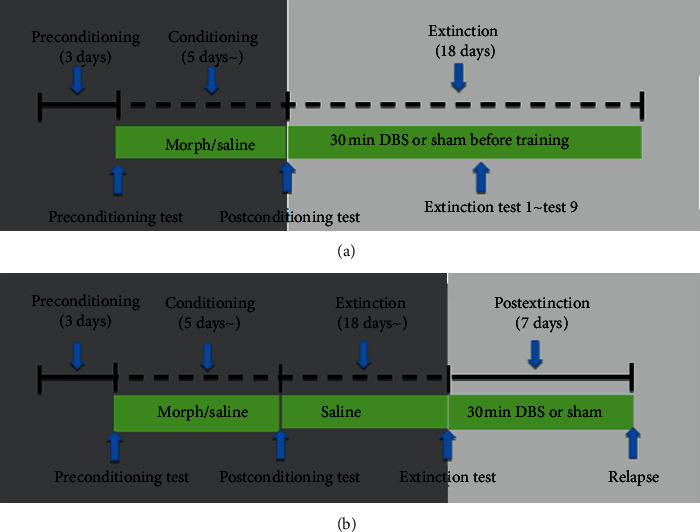
The timetable for the experimental process . (a) Extinction-stimulation group. (b) Extinction-stimulation group.

Forty SD rats were divided into extinction stimulation group (*n* = 20) and postextinction stimulation group (*n* = 20) after the establishment of the CPP model. Each group was divided into two subgroups, including the experimental group (*n* = 10) and control group (*n* = 10). In the extinction stimulation group, the experimental group received HFS before the extinction induction, while the control group received pseudostimulation. In total, nine stimulations were performed. In the postextinction stimulation group, the experimental group was treated with HFS during postextinction, while the control group received pseudostimulation. After 7 days of continuous stimulation or pseudostimulation, small doses of morphine were administrated to induce relapse after the last stimulation. The experimental process is shown in Figure 2.

### 2.6. Tissue Staining

At the end of the experiment, three rats in each group were anesthetized with 4% pentobarbital sodium (5 ml/kg) and then continuously perfused with 4% paraformaldehyde. The brain was quickly removed and was fixed for 24 h in 4% paraformaldehyde and then put into 30% sucrose solution. Brain slices with a thickness of 30 *μ*m were cut using an icy slice machine at −20°C. The brain slices containing the location of implanted electrodes were selected and pasted on glass slides. The slides were stained with 0.2% hematoxylin. The OLYMPUS microscope camera system and the CELLSENS microscopic image analysis software were used for image analysis to confirm whether the electrode was placed correctly.

### 2.7. Statistical Analysis

The experimental results are expressed as mean ± standard deviation. The paired *t*-test was used in the comparison within the same group. The two-sample independent *t*-test was applied to compare the relapse scores between the two groups. Statistical analysis was performed using SPSS19.0 software, and *P* < 0.05 was considered to be statistically different. The figures were produced with GraphPad Prism 5.

## 3. Results

### 3.1. The Establishment of Morphine-Induced CPP Model

After 5 days of treatment, the average postconditioning CPP score of the rats was 603.2 s ± 80.80 s and the average preconditioning CPP score was 371.1 s ± 60.84 s. Thus, the morphine-induced training obviously prolonged the stay time in the drug box (*P* < 0.0001), indicating the successful establishment of the CPP model.

### 3.2. The Effect of High-Frequency Electric Stimulation on the Extinction of Rat

Both the experiment group and control group received the extinction induction. The experimental group received HFS before the extinction induction, and the control group was treated with pseudostimulation, each stimulation lasting 30 min. The extinction CPP score was tested on the next day and compared with preconditioning CPP score and postconditioning CPP score, respectively, using paired *t* tests. Totally, nine electrical stimulations were treated in both groups.

**Figure 3:**
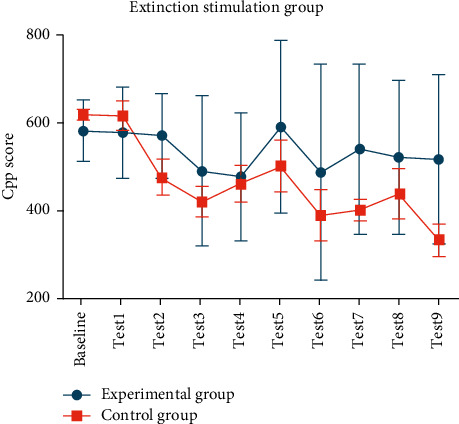
The effect of HFS on the extinction phase of morphine CPP in rats: the experimental group did not regress successfully during the 9 electrical stimulations; control group got successful induction of extinction after third stimulation, which showed the inhibition of extinction in the experimental group.

The results (Figure 3) showed the following:

1. After third stimulation, the average extinction CPP score in control group was 466.66 s ± 157.17 s, which showed no significant statistical difference to preconditioning CPP score (377.38 s ± 66.76 s, *P*=0.0871), but is significantly different from postconditioning CPP score (620.07 s ± 88.21 s, *P*=0.0154). The results indicate the successful induction of extinction.
2. After HFS, the experimental group did not regress successfully during the 9 electrical stimulations.
3. Comparing the extinction CPP score in experimental group and the control group, the difference between the two groups was statistically significant (*P*=0.0212), indicating the inhibition of extinction in the experimental group.

### 3.3. The Effect of HFS on Kindling Relapse during Postextinction Stages

The extinction was induced after the establishment of CPP model in the postextinction stimulation group. There was no significant difference between the extinction CPP score (456.4 s ± 148.8 s) and preconditioning CPP score (353.9 s ± 84.33 s), while a significant difference was shown between extinction CPP score and postconditioning CPP score, indicating the successful induction of extinction in rats.

**Figure 4:**
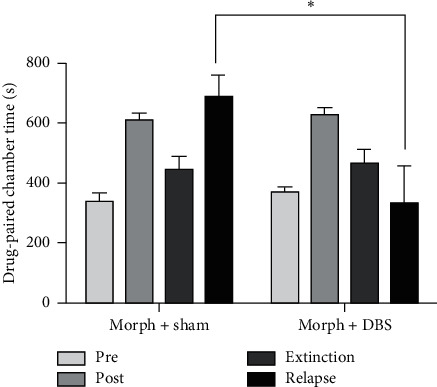
The effect of HFS on kindling relapse during postextinction stages. Morph + sham: control group; morph + DBS: experimental group; pre = preconditioning CPP score; post = postconditioning CPP score; extinction = extinction CPP score; relapse = relapse CPP score. ^∗^ indicates *P* < 0.05. The results suggest that high-frequency DBS stimulation during withdrawal period will inhibit the rats' relapse behavior.

The rats with extinction were randomly divided into the experimental group and the control group. The experimental group was treated with HFS; the control group was given pseudostimulation for 7 days. A relapse test was performed in two groups to obtain the relapse CPP score, respectively, within 24 hours of the last stimulation (Figure 4).

1. There is no significant difference between relapse CPP score (330.1 s ± 212.6 s) and preconditioning CPP score (372.2 s ± 52.75 s) in the experimental group.
2. The relapse CPP score (684.2 s ± 230.2 s) was significantly different from the preconditioning CPP score (335.6 s ± 107.2 s) in the control group.
3. Relapse CPP score in the experimental group (330.1 s ± 212.6 s) was significantly different from the relapse CPP score in the control group (684.2 s ± 230.2 s) (*P*=0.029), suggesting that HFS can significantly inhibit the relapse induced by morphine.

### 3.4. The Confirmation of Location of DBS Electrode

**Figure 5:**
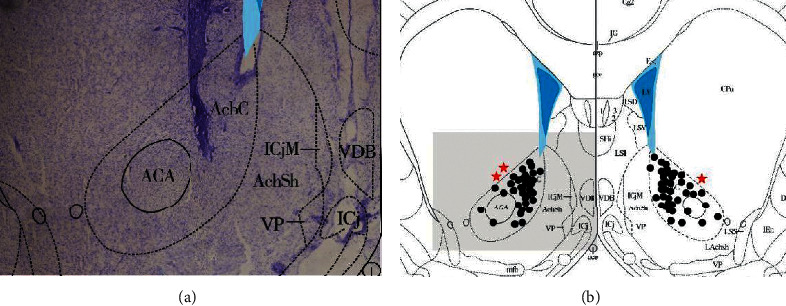
The confirmation of location of DBS electrode. (a) The electrode tip is precisely located in nucleus accumbens. AcbC represents nucleus accumbens; AchSh represents the shell of nucleus accumbens; ACA represents the former union; ICjM represents the major part of parahippocampal gyrus; VDB represents diagonal band vertical arm nucleus; VP represents ventral pallidum; ICj represents parahippocampal gyrus. (b) Bregma 1.80 mm plane is the electrode coordinate plane, and the shaded area is the tissue section range of 5(a). The black points are in the NAc, and the red stars are the inaccurate points.

All the rats in the experiment were executed through decapitation, and the brains were removed to observe whether the tip of the DBS electrode was located in the nucleus accumbens. As shown in Figure 5, the position of the electrode tip is located in the nucleus accumbens.

## 4. Discussion

In the established morphine-induced CPP model, the effects of HFS of bilateral nucleus accumbens on the drug seeking behavior of rats were observed during the extinction phase and postextinction phase. The results showed that HFS inhibits extinction during the extinction stage in morphine-induced CPP in rats. In addition, HFS can significantly inhibit morphine-induced relapse after extinction of addictive behavior.

DBS stimulation may enhance and consolidate memory by affecting memory-related neural circuits [28]. The nature of drug seeking behavior is a pathological memory based on drug-induced gene expression and changes of synaptic plasticity. The formation and consolidation of memory is a process of gradual reconstruction from the short-term changes of synaptic plasticity in local neurons to long-term changes of connectivity between different brain regions. Under certain conditions, the memory that has been formed can be reactivated and entered into an unstable state. In this process, memory can be remodeled and even updated [29, 30]. The memory of reward conforms to the process of formation and extinction of normal memory. However, the long-term effects of addictive drugs caused changes in the plasticity of synapses in the addiction-related brain regions, resulting in pathological alterations in the number, density, and morphology of synapses [31–34], especially changing the long-term potentiation (LTP) and the long-term depression (LTD). Finally, the remodeling of the addiction-related brain regions transformed the short-term reward memory into long-term memory, which may usurp or utilize the normal memory-related neural circuits. These changes may cause persistent and strong addiction memory, as well as long-term resistance to addiction extinction [35]. Liu et al. found that HFS could prevent morphine-induced CPP by attenuating morphine-induced preference enhancement [19]. We found that HFS could delay the extinction process in the stage of drug seeking behavior withdrawal, and HFS during the postextinction period inhibited relapse behavior. To sum up, we think that HFS of the NAc of the animal model in different periods of drug seeking behavior may strengthen the memory during the stimulation through some mechanism, making it not easy to be activated and remolded.

HFS has been shown to induce a “distant excitation” [36–40], which increases electrical activity in nearby axonal projections. HFS suppressed firing of neuronal populations surrounding the stimulation electrode, which led to functional target inactivation. After DBS, physiological neuronal firing is substituted by an artificial tonic pattern, which drives axonal pathways near the electrodes, leading to changes in neuronal firing rate and pattern in structures projecting to or receiving projections from the target [36]. The general understanding of the reward circuitry underlying addiction begins with the VTA, and a major reward-related output of VTA is NAc. Dopamine (DA) can exert its effects via activation of DA receptors located on medium spiny neurons (MSNs). HFS induced artificial tonic pattern possibly affecting MSNs in NAc and their output projection, changing the LTP and LTD, finally influencing the whole memory-related neural circuits and making the circuits difficult to be activated and remodeled.

Our results suggest that the extinction of the addictive behavior can be prolonged by HFS treatment during the extinction phase, and HFS can effectively inhibit the relapse behavior after the addictive behavior is completely eliminated. The results suggest that DBS can be used to treat relapse of addiction after withdrawal of addictive behavior. However, the extinction period may be the optimal period for DBS intervention for drug addiction. The addictive memory is closely related to the circumstances of drug taking, and these two components are usually integrated into a unified memory. The NAc core appears to be critical for assigning motivational value to discrete stimuli associated with reward or aversion and particularly updating these values as circumstances change, which induces the activation of the nucleus accumbens to induce addictive relapse behavior [40, 41]. Hamilton et al. found that during addiction withdrawal period, HFS of NAc was more effective than low-frequency stimulation in inhibiting cocaine-induced relapse [14]. In the present study, we found that though the extinction was significantly prolonged by HFS, the relapse behavior was also inhibited after postextinction treated by HFS. It has been discussed above that DBS may influence the memory-related neural circuits, resulting in consolidation and enhancement of memory. In conclusion, the optimal protocol for HFS for addiction treatment was to induce extinction and simultaneously give HFS to NAc. During the slow regression of addictive behavior, the HFS of the NAc will consolidate and strengthen the addictive behavior and environmental conditions in the memory-associated neural circuits. Once the addiction behavior is completely eliminated, the animals may be less likely to relapse during postextinction.

NAc-DBS may be widely accepted as a new treatment for drug addiction. In this study, the effect of NAc-DBS on the addictive behavior of rats in different periods of addiction was studied to provide evidence and support for the appropriate period of clinical use of DBS in the treatment of drug addiction. At the same time, by observing the effect of DBS on addiction behavior in different periods of addiction, the hypothesis of memory enhancement of DBS was proposed for rational interpretation of the experimental findings. However, it should be pointed out that the results and hypotheses may only apply to morphine-induced drug addiction behavior. The strengthening effect of memory after DBS and its related mechanisms still need further clinical and basic research studies.

## Data Availability

Data are available on request from the corresponding author.
